# Genetic and Epigenetic Regulation of Lipoxygenase Pathways and Reverse Cholesterol Transport in Atherogenesis

**DOI:** 10.3390/genes13081474

**Published:** 2022-08-18

**Authors:** Stanislav Kotlyarov

**Affiliations:** Department of Nursing, Ryazan State Medical University, 390026 Ryazan, Russia; skmr1@yandex.ru

**Keywords:** atherosclerosis, genes, polymorphism, epigenetics, microRNA, lncRNAs, inflammation, lipid mediators

## Abstract

Atherosclerosis is one of the most important medical and social problems of modern society. Atherosclerosis causes a large number of hospitalizations, disability, and mortality. A considerable amount of evidence suggests that inflammation is one of the key links in the pathogenesis of atherosclerosis. Inflammation in the vascular wall has extensive cross-linkages with lipid metabolism, and lipid mediators act as a central link in the regulation of inflammation in the vascular wall. Data on the role of genetics and epigenetic factors in the development of atherosclerosis are of great interest. A growing body of evidence is strengthening the understanding of the significance of gene polymorphism, as well as gene expression dysregulation involved in cross-links between lipid metabolism and the innate immune system. A better understanding of the genetic basis and molecular mechanisms of disease pathogenesis is an important step towards solving the problems of its early diagnosis and treatment.

## 1. Introduction

Despite the modern development of medicine, atherosclerosis remains one of the most important medical and social problems. Its significance is well demonstrated by negative statistics on the number of cases of temporary and permanent disability and mortality from atherosclerotic cardiovascular diseases [1]. Coronary heart disease, cerebral stroke, and peripheral arterial disease are frequent reasons for seeking medical care, hospitalizations, and are associated with a large number of unresolved diagnostic and therapeutic issues in clinical practice [2,3]. Epidemiological data indicate that the problem of atherosclerosis is urgent for many countries of the world, and atherosclerosis is a serious economic burden for both individual patients and healthcare systems [4,5,6].

A frequent situation in real clinical practice is untimely application of patients for medical care, which leads to late diagnosis when there are already serious clinical manifestations of the disease. These and other data intensify the attention on the discussion about the necessity to find new methods of atherosclerosis diagnostics, which would allow to estimate individual predisposition and individual trajectories of its development and progression.

The etiology and pathogenesis of atherosclerosis have long been the subject of close attention of clinicians and researchers. The results of numerous studies have allowed the proposal of a number of theories about atherogenesis, which assumed the action of many factors and the involvement of various mechanisms [7,8]. A number of modifiable factors, such as smoking and dyslipidemia, are of great clinical importance. Lipids are thought to occupy a special place in the pathogenesis of atherosclerosis. They both act as a substrate for the morphological basis of atherosclerosis and are actively involved in cross-linkages with the innate immune system [9].

Accumulating evidence suggests that the development and progression of atherosclerosis are associated with impaired mechanisms of the innate immune system [10,11]. Indeed, macrophages, which perform many functions of the innate immune system, are actively involved in the pathogenesis of atherosclerosis [12]. It is also known that there are close cross-links between the innate immune system and cellular metabolic processes [13].

Moreover, there is a growing understanding that, in addition to risk factors with a systemic mechanism of action, local factors may also be associated with the development and progression of atherosclerosis. Hemodynamics are an important local factor, which influences the distribution pattern of atherosclerotic lesions in the vascular bed [9].

The variety of factors associated with the development of atherosclerosis increases the attention of clinicians and researchers to the identification of genes that may be involved in atherogenesis [14,15,16]. Interest in unraveling the genetic basis of atherosclerosis is increasing due to recent advances in molecular and genetic research methods, as well as improvements in bioinformatics data analysis methods.

Thus, the search for new keys to understanding the pathogenesis of atherosclerosis is an important step toward identifying new targets in the diagnosis and treatment of atherosclerosis.

## 2. Genetic Contribution of the Lipoxygenase Pathway

Lipid metabolism and its disorders occupy a special place in the pathogenesis of atherosclerosis, which has been shown in numerous studies and became the basis for supporting the lipid theory of atherogenesis. It should be noted that the content of this theory finds new meaning through the understanding that lipids are not just substrates for the morphological basis of atherosclerosis, but are also mediators of inflammation and inflammation resolution [17]. Lipid mediators contribute significantly to the pathogenesis of atherosclerosis. Leukotrienes, products of the enzymatic conversion of arachidonic acid, are involved in the maintenance of inflammation [18,19]. At the same time, specialized pro-resolving mediators (SPM) belonging to the groups of lipoxins, resolvins, maresins, and protectins provide resolution of inflammation. They are formed enzymatically from ω-3 and ω-6 polyunsaturated fatty acids (PUFAs), such as eicosapentaenoic, docosahexaenoic, and arachidonic fatty acids [20].

**Figure 1:**
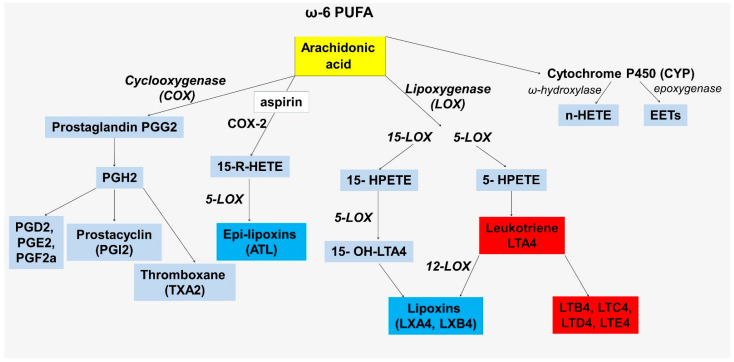
Scheme of the formation of lipid metabolites involved in the regulation of inflammation from arachidonic acid.

Arachidonic acid is at the crossroads of inflammation and inflammation resolution, acting as a substrate for the formation of lipid mediators [20]. Arachidonic acid is released from plasma membranes by phospholipase A2 and can then be metabolized through the lipoxygenase (LOXs), cyclooxygenase (COXs), and cytochrome P450 enzymes (Figure 1) [21].

All facets of the delicate balance between the maintenance and resolution of inflammation involving lipid mediators have yet to be explored, but the role of imbalances between the production of pro- and anti-inflammatory mediators as an important link in the pathogenesis of atherosclerosis is already known. The complexity of this balance in addition to the common substrate, arachidonic acid, is also in the commonality of a key enzyme required for one of the stages of biosynthesis—5-lipoxygenase (5-LOX). This member of the lipoxygenase family is encoded by the *ALOX5* gene. In total, human lipoxygenases include 6 different LOX isoforms that are encoded by 6 functional LOX genes, including *ALOX5*, *ALOX12*, *ALOX12B*, *ALOX15*, *ALOX15B*, and *ALOXE3* [22,23]. *ALOX5*, along with *ALOX15*, are best known for their involvement in atherogenesis [22]. The enzymes encoded by these genes are at the intersection of proinflammatory and pro-resolving pathways (Figure 1). The importance of 5-LOX in these processes is thought to be determined by its cellular localization. The nuclear localization of 5-LOX makes the enzyme a key link in leukotriene biosynthesis because of its proximity to leukotriene A4 hydrolase [24,25,26]. In this pathway, 5-LOX catalyzes the dioxygenation of arachidonic acid to 5S-HpETE followed by the conversion of 5S-HpETE to leukotriene A4 (LTA4). LTA4 is hydrolyzed to leukotriene B4 (LTB4) by the leukotriene A-4 hydrolase, which is encoded by the LTA4H gene. In addition, LTA4 can also be conjugated by LTC4 synthase (*LTC4S*) to form a series of three cysteinyl leukotrienes (LTC4, LTD4, LTE4) [27].

In non-nuclear localization, 5-LOX is more closely related to 12/15-LOX, thus promoting the conversion of arachidonic acid to pro-resolving lipoxin LXA4 and docosahexaenoic acid to resolvin (Rv)D1. These cross-links are important in balancing the maintenance and resolution of inflammation, but they can be disrupted in atherosclerosis. In addition, these data highlight the dual role of 5-LOX in atherogenesis, which is of clinical and research interest.

The *ALOX5*-related leukotriene signaling pathway is an evolutionarily ancient inflammatory mechanism found in all higher vertebrates [28,29]. The expression of 5-LOX in lipopolysaccharide-stimulated monocytes is regulated at the level of transcription through Toll-like receptor 4 (TLR4)/Akt-mediated activation of the Sp1 and NF-κB pathways [30].

LTB4, is an important participant in inflammation in the vascular wall. It exhibits multiple proinflammatory functions, is involved in the expression of endothelial adhesion molecules, and promotes leukocyte transmigration and activation. In addition, LTB4 enhances monocyte transformation into foam cells by enhancing CD36 expression and fatty acid accumulation [31]. Through these and other functions, LTB4 has been shown to be involved in various stages of atherosclerosis progression. In addition, its action is associated with the development of a vulnerable plaque phenotype and negative clinical outcome [32].

High levels of 5-LOX expression have been found in carotid artery walls affected by atherosclerosis and correlate with signs of plaque instability [33]. 5-LOX is expressed in many cells, such as macrophages, dendritic cells, mast cells, and neutrophils. The number of cells expressing 5-LOX increases in advanced lesions [34]. Increased 5-LOX expression in symptomatic plaques corresponds to increased LTB4 production and biosynthesis of matrix metalloproteinase-2 (MMP-2) and MMP-9, which are associated with acute ischemic syndromes [35]. It was also shown that patients with a history of recent clinical events, such as transient cerebral ischemic attack or minor stroke, had higher levels of 5-LOX mRNA determined [33]. In an experimental mouse model of atherosclerosis, blockade of the LTB4 receptor reduces lesion progression by inhibiting monocyte recruitment [36]. Simultaneously with high levels of LTB4, SPM levels, especially RvD1, and the ratio of SPM to LTB4 are reduced in the region of unstable atherosclerotic plaques [37].

These and other data have increased attention to *ALOX5* as a key participant in the leukotriene pathway. *ALOX5* has been identified as a gene that contributes to susceptibility to atherosclerosis in mice [38]. Genetic deficiency of *ALOX5* in mice results in reduced development of atherosclerosis, despite increased lipid levels, fat mass, elevated glucose levels, and low fasting insulin levels [38,39]. Lack of *ALOX5* in mice led to increased obesity with age and a number of metabolic defects, including fatty liver, as well as decreased production of inflammatory resolution mediators by macrophages [40]. Aging in *ALOX5*-deficient mice causes macrophage dysfunction with an increased proinflammatory phenotype, downregulation of the formyl peptide receptor type 2 (FPR2) in the heart after myocardial infarction [40]. Deficiency of 5-LOX in mice attenuated aortic aneurysm formation, which was accompanied by decreased MMP-2 activity and decreased plasma macrophage inflammatory protein-1 alpha (MIP-1α). However, these processes had a minimal effect on the formation of lipid-rich lesions [41]. In another study, genetic inhibition of 5-LOX did not confirm a role for the leukotriene pathway in the development of atherosclerotic lesions in *ApoE*-deficient mice [42]. A triple mutation of the *Alox5* gene in mice reduced the biosynthetic ability of the enzyme to proinflammatory leukotrienes in favor of 13-HODE formation from linoleic acid. These mice cannot synthesize proinflammatory leukotrienes but have increased body weight and significantly increased levels of 13-HODE in adipose tissue [43]. Interestingly, 13-HODE is a potent activator of PPARγ and also activates PPARα, resulting in increased ABCA1 expression and enhanced cholesterol efflux from macrophages [44,45,46]. Dual deficiency of 12/15-LOX and 5-LOX in macrophages alters arachidonic acid metabolism and attenuates peritonitis and atherosclerosis in *ApoE* knockout mice [47].

Interestingly, 5-LOX deficiency or inhibition increases the mortality of mice after experimental myocardial infarction due to healing defects, which is associated with impaired inflammatory cell function and decreased migration ability of 5-LOX^−/−^ fibroblasts [48]. It was also shown that in experimental myocardial infarction in 5-lipoxygenase-deficient mice there was an increase in neutrophil infiltration and tumor necrosis factor (TNF) expression. This indicates the effect of 5-LOX inhibition on the postischemic inflammatory response [49]. Targeting RNAi at *ALOX5* resulted in a 19% decrease in *ALOX5* expression in myocardial tissue and a 3.8-fold reduction in infarct size in experiments in a rat model of myocardial ischemia-reperfusion [50]. This is consistent with the evidence that siRNA-mediated silencing of *ALOX5* reduces neonatal cardiomyocyte necrosis during anoxia-reoxygenation [51]. It is important to respond that mice with *Alox5* gene knockout are more susceptible to complications of experimental infection and exhibit a worsened inflammatory response. Thus, experiments with animals have shown different effects of *ALOX5* deficiency.

Evaluation of the clinical significance of the gene polymorphism also demonstrated different results. The 5-LOX genotype variants have been shown to be associated with in-creased intima-media thickness and to identify a subpopulation with increased atherosclerosis [52]. In addition, *ALOX5* polymorphisms independently predict severe carotid artery disease [53]. However, in other studies, 5-lipoxygenase gene polymorphisms have not been associated with atherosclerosis or coronary heart disease and risk of myocardial infarction [54,55,56]. These and other data support an ongoing debate regarding the contribution of *ALOX5* gene polymorphisms to atherogenesis and its clinically manifest forms.

Interestingly, *ALOX5* gene variants affect eicosanoid production in response to fish oil supplementation [57]. Dietary ω-6 PUFAs have also been found to promote and marine ω-3 PUFAs to inhibit leukotriene-mediated inflammation [52,58]. Despite these findings, the interactions between *ALOX5* (rs59439148) polymorphisms and arachidonic and eicosapentaenoic acid content in adipose tissue had no effect on the risk of myocardial infarction in middle-aged men and women [59].

Genetic variants in another member of the leukotriene pathway, *ALOX5AP*, may be associated with the pathogenesis of both myocardial infarction and stroke by increasing leukotriene production and increasing inflammation in the arterial wall [60,61,62]. The *ALOX5AP* gene encodes the 5-lipoxygenase activating protein (FLAP), which anchors 5-LOX to the membrane and also binds arachidonic acid and may play an important role in transferring arachidonic acid to 5-LOX. FLAP is a nuclear membrane protein and is required for the synthesis of both leukotrienes and LXA4/RvD1 [63]. Analysis of angiographic data showed a possible modest role for *ALOX5AP* in the development of atheroma, but not on its clinically manifest forms, such as myocardial infarction [64]. Genetic variability in ALOX5AP has also been associated with myocardial infarction in the German population [65]. In addition, genetic variation in the *ALOX5AP* gene contributes to the risk of coronary heart disease (CHD) in patients with familial hypercholesterolemia [66]. It should be noted that analysis of *ALOX5AP* polymorphisms showed different evidence for associations with the risk of ischemic stroke [67,68,69,70,71,72].

Another member of the leukotriene pathway, the *LTA4H* gene encoding leukotriene A4 hydrolase, confers a moderate risk of myocardial infarction [73]. Four single nucleotide polymorphisms (SNPs) in the LTA4H gene showed a significant association with levels of LTB4, which acts as a link to the risk of CHD [74]. In addition, the rs2540489 polymorphism in the *LTA4H* gene appears to be associated with ischemic stroke of the large arteries [75].

15-LOX, encoded by the *ALOX15* gene, is a key enzyme in the synthesis of SPM [76]. 15-LOX can convert arachidonic acid to 15-hydroperoxycozatetraenoic acid (15-HPETE), which is metabolized to 15-hydroxycozatetraenoic acid (15-HETE), which is a vasoconstrictor [77]. In addition, lipoxygenases encoded by the *ALOX15* and *ALOX15B* genes may participate in atherogenesis through their ability to oxidize esterified PUFAs and cholesterol esters in plasma membranes and lipoproteins and participate in foam cell formation [76,78,79,80].

*ALOX15* suppresses the TNF-α, IL-1β/NF-κB, and IL-6/STAT3 signaling pathways and also promotes the formation of resolvins, which are some of the most important lipid mediators involved in resolving inflammation [81]. None of the *ALOX15* polymorphisms has been associated with myocardial infarction, but the rare *ALOX15* haplotype has shown a significant protective effect on the risk of myocardial infarction [82]. The c.-292C > T promoter polymorphism increases reticulocyte-type 15-lipoxygenase-1 activity and may be atheroprotective through the production of 15(S)-HETE and its pro-resolving metabolites, lipoxins [83].

*ALOX15B* gene polymorphisms may also be associated with coronary heart disease [84]. *Alox15b* knockdown in mice led to a decrease in atherosclerosis as measured by plaque area, as well as to a decrease in the severity of inflammation, which confirms the atherogenic role of *ALOX15B* [85]. It should be noted that *Alox15b* and the enzyme it encodes in mice and humans have several fundamental differences.

Interestingly, *ALOX15B* and, to a lesser extent, *ALOX15* are involved in the regulation of cholesterol levels in macrophages via SREBP-2 [86]. It has previously been shown that overexpression of human *ALOX15* in RAW macrophages contributes to increased ABCA1-mediated cholesterol efflux [87]. These data reinforce the understanding of the significance of cross-links between lipid transport and inflammation.

Of note, the combination of leukotriene pathway gene polymorphisms may also be important. *ALOX12, ALOX5*, and *ALOX5AP* polymorphisms are genetically associated with subclinical atherosclerosis and with disease biomarkers in families with type 2 diabetes [88]. Genetic variations in the oxidative stress-related genes *ALOX5*, *ALOX5AP*, and *MPO* have also been shown to modulate susceptibility to ischemic stroke through the main effects and epistatic interactions [89]. However, the Athero-Express Genomics study showed that variants in ALOX5, ALOX5AP, and LTA4H were not associated with atherosclerotic plaque phenotypes, suggesting a limited role of their common variants on the morphology of progressive atherosclerotic plaque [90].

Another interesting area of research is assessing the links between mitochondrial dysfunction, inflammation, and oxidative stress [91]. Moreover, PUFA metabolic products can affect mitochondrial function [92]. Research into the genetic regulation of these pathways is of clinical interest. 12-HETE is one of the arachidonic acid metabolites formed in the lipoxygenase pathway, known for its role in mitochondrial dysfunction [93]. *ALOX12* gene polymorphisms are associated with the development of atherosclerosis, cardiovascular disease, and 12-HETE levels [94,95,96].

Thus, the potential importance of the lipoxygenase pathway in the pathogenesis of atherosclerosis is beyond doubt. At the same time, the significance of polymorphism of genes encoding these enzymes still remains a topic for research and discussion, which requires new data and their analysis. It should be noted that 5-LOX is located at the crossroads of inflammation activation and resolution pathways, so the interpretation of the enzyme’s function in atherogenesis remains a matter of debate.

## 3. Genetic Contribution to Reverse Cholesterol Transport

Reverse cholesterol transport is an important homeostatic pathway that ensures the removal of excess cholesterol from peripheral tissues (Table 1). This atheroprotective mechanism involves the participation of high-density lipoproteins (HDL), which have an important transport function and through it demonstrate involvement in innate immunity [97]. HDL formation is linked to the lipid export function of ABCA1, a member of a family of membrane proteins that transport chemically diverse substrates across the lipid bilayer of cell membranes [98,99]. Expression of ABCA1 is regulated at both the transcriptional and post-transcriptional levels (Table 1) [100,101].

ABCA1 exports cholesterol from macrophages, saturating the nascent HDL particles, which are then further lipidized by another member of this family, ABCG1 [97]. In this regard, ABCA1 is considered to be a key participant in HDL biogenesis. Mutations in the *ABCA1* gene are known to cause a rare genetic Tangier disease, which is characterized by a significant decrease in HDL levels and an increased incidence of cardiovascular disease [108,109,110]. In addition to Tangier disease, variations and changes in the ABCA1 gene increase the risk of atherosclerosis [111,112], familial hypercholesterolemia [113], coronary heart disease [114], including myocardial infarction [115,116] and ischemic stroke [112,117], and can be considered as a prognostic biomarker [118,119]. *ABCA1* gene polymorphisms modulate HDL cholesterol levels and, thus, may influence cardiovascular risk in the general population [120,121,122].

In addition, the r219k polymorphism of the *ABCA1* gene may influence the hypolipidemic effect of pravastatin in patients with coronary heart disease [123,124]. In addition to an atherogenic effect, the AA genotype of the G1051A polymorphism is associated with higher HDL cholesterol levels and a lower prevalence of coronary artery calcification [125]. These and other data reinforce the understanding of the significance of genetic variation in *ABCA1* [126].

Interestingly, despite the atheroprotective role of *Abca1*, it has been shown to adversely affect cardiac function after myocardial infarction in mice. *Abca1*^−/−^ mice had a smaller infarct size after coronary artery ligation and increased T- and B-lymphocytes [127].

Importantly, the lipid-transporting function of ABCA1 makes it an important participant in the innate immune system through regulation of cholesterol content in macrophages [128]. The removal of excess cholesterol has an anti-inflammatory effect on macrophages [129]. This effect is mediated by modification of the biophysical properties of the plasma membrane and changes in the function of membrane proteins, such as TLR4 and their signaling pathways, direct cholesterol-protein interactions and other mechanisms [130].

In addition, methylation levels of the ABCA1 promoter may be associated with inflammation and the development of premature coronary heart disease. It was shown that *ABCA1* promoter methylation levels were positively associated with serum inflammatory factors (CRP, IL-1β), circulating free DNA/neutrophil extracellular traps (cfDNA/NETs), suggesting a role for inflammatory responses in premature coronary heart disease [131]. Higher levels of *ABCA1* DNA methylation in older men have also been shown previously to be associated with low levels of high-density lipoprotein cholesterol [132].

Thus, lipids show different cross-linkages with inflammation, which has implications for atherogenesis. In addition, key players in these processes also show involvement in different processes, both pro- and anti-atherogenic, requiring a more detailed study of their complex reciprocal influence rather than of individual mechanisms.

## 4. Role of microRNAs

Numerous recent studies highlight the importance of non-coding microRNAs (mi-croRNA, miRNA) in the pathogenesis of several diseases. Small non-coding RNA molecules are 18–25 nucleotides long (average length is 22), and take part in transcriptional and post-transcriptional regulation of gene expression. Because of this they are involved in the regulation of many biological processes, such as cell differentiation, growth, proliferation and apoptosis. MicroRNAs are highly conserved among eukaryotes, representing a part of evolutionarily ancient component of gene expression regulation system.

Biosynthesis of microRNAs is now described in detail and involves a series of sequential processes, including the formation of primary miRNA transcripts (pri-miRNAs) by RNA polymerase II, their subsequent cleavage in the nucleus by Drosha endonuclease to form pre-miRNAs. After transport into the cytoplasm, they are subsequently processed by a multiprotein complex, including Dicer [133,134].

MicroRNAs are involved in the regulation of gene expression at the post-transcriptional level by binding to the 3′-untranslated regions (UTRs) of target mRNAs [135]. A single microRNA can bind to target sequences in several mRNAs, usually leading to mRNA degradation or translation inhibition. Thus, microRNAs exert post-transcriptional control in various signaling pathways.

**Figure 2:**
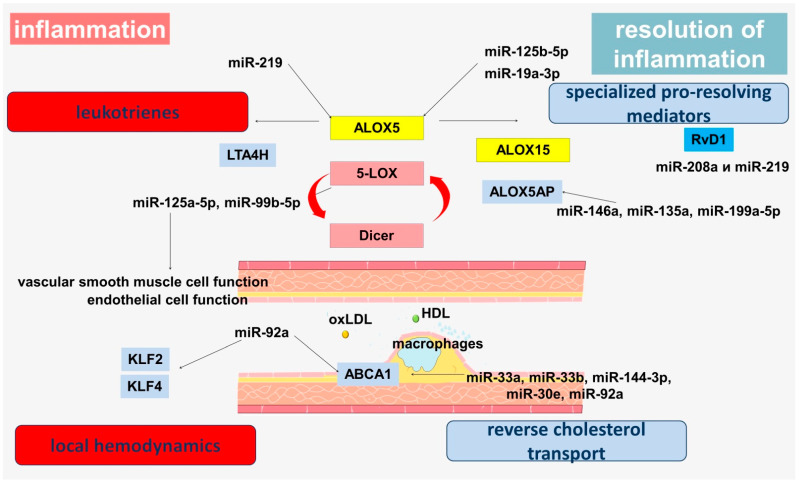
MicroRNA involvement in the pathogenesis of atherosclerosis.

A growing body of evidence strengthens the understanding of the importance of microRNAs in the pathogenesis of atherosclerosis. MicroRNAs are involved in the modulation of the leukotriene pathway through the regulation of 5-LOX (Figure 2). The 5-LOX has been shown to target miR-19a-3p and miR-125b-5p. Inhibition of both microRNAs by antagomirs resulted in a significant increase in 5-LOX protein expression in the myeloid cell line [136]. Another study showed downregulation of miRNA-125b-5p and miR-193a-3p in aortic aneurysm tissues, leading to increased leukotriene production, increased inflammation and aortic wall damage through upregulation of the *ALOX5* gene [137]. The involvement of miR-193a-3p in the regulation of vascular smooth muscle cells (VSMCs) proliferation and migration has also been shown, allowing it to be considered as a regulator of phenotypic switching in VSMCs [138].

MiR-125a/b-5p in vascular endothelial cells inhibits the expression of endothelin-1 (ET-1), which is a potent vasoconstrictor peptide. It has been shown that miR-125a/b-5p can suppress oxLDL-induced ET-1 expression by targeting the 3′-untranslated mRNA region of preproendothelin-1 (preproET-1) [139]. These findings demonstrate the cross-talk between vascular inflammation and hemodynamics.

Clinical evaluation of microRNA significance showed that plasma expression levels of miR-125b-5p and miR-30d-5p were higher in patients with acute coronary syndrome compared with healthy controls. In addition, miR-125b-5p levels were associated with 6-month cardiovascular events in patients with acute myocardial infarction [140].

Another microRNA, miR-219 regulates 5-LOX protein levels and LTB4 generation [141,142]. Overexpression of miR-219 has been shown to result in a significant reduction in 5-LOX and leukotriene production [141]. Interestingly, miR-219-2 has been identified as a regulator of 5-LOX in macrophages, which can reduce pro-inflammatory LTB4 production and increase pro-resolving mediators, including protectin D1 (PD1) [143]. In addition, RvD1 activates its receptors on human macrophages to regulate miR-219-5p, which is formed from pre-miRs miR-219-1 and miR-219-2 [143]. The anti-inflammatory effects of RvD1 are mediated in part by microRNAs including miR-21, miR-146b, miR-208a and miR-219 [141]. RvD1 administration significantly up-regulates miR-208a and miR-219 in exudates isolated from ALX/FPR2 transgenic mice [144]. In human macrophages, miR-219, miR-208a, miR-146b and miR-21 have been identified as RvD1-GPCR-regulated microRNAs [144]. Thus, miR-219 is an important regulator of the inflammatory signaling pathway.

Interestingly, 5-LOX can modulate miRNA processing in monocytic cells. It has been shown that 5-LOX can interact with human Dicer [133,145,146]. Dicer, which is involved in microRNA biogenesis, is known to interact with cellular proteins through its N-terminal domain. Thus, Dicer has been shown to interact with 5-LOX. On the one hand, the C-terminal of human Dicer (5-lipoxygenase binding domain or 5LObd) enhances the enzymatic activity of 5-LOX, and, on the other hand, 5-LOX modifies the pre-miRNA processing activity of Dicer. Thus, the processing of specific microRNAs can be regulated by the interaction between 5-LOX and Dicer. This relationship represents an interesting cross-sectional mechanism between inflammation and miRNA-driven regulation of gene expression [145].

It was shown that 5-LOX promotes the transcription of the miR-99b/let-7e/miR-125a cluster. Moreover, 5-LOX–Dicer interaction led to a decrease in pre-let-7e processing and an increase in miR-125a-5p and miR-99b-5p levels without concomitant changes in let-7e levels [133]. let-7e is an important regulator of endothelial function and acts as a proinflammatory mediator involved in the regulation of the NF-κB pathway through inhibition of its target gene (IkBβ) expression [147]. It has been shown that miR-125a-5p has protective functions for endothelial cells and can be transported by endothelial cells released microvesicles, enhancing endothelial cell survival and angiogenic function through modulation of PI3K/Akt/eNOS and caspase-3 pathway expression [148]. It has previously been shown that miR-125a-5p can counteract the effects of ox-LDL on various endothelial cell functions through regulation of the EGFR/ERK/p38 MAPK and PI3K/Akt/eNOS pathways and cleavage of caspase-3, ICAM-1, and VCAM-1 expression [149]. MiR-125a-5p also inhibits NLRP3 expression by targeting CCL4 in human VSMCs treated with ox-LDL [150]. MiR-125a and miR-125b may play a role in regulating the TLR/MyD88/NF-κB axis [151,152]. Thus, miR-125a-5p levels are decreased in atherosclerotic plaques of patients with coronary atherosclerosis [153].

FLAP can also be regulated by microRNAs (Figure 2). MiR-146a suppresses FLAP expression and LTB4 production and regulates COX-2 in lung cancer cells [154]. In addition, miR-146a is a target for reducing inflammation in patients with CHD because it targets interleukin-1 receptor-associated kinase 1 (IRAK-1) and tumor necrosis factor receptor associated factor 6 (TRAF-6), which leads to inhibition of NF-κB via TLR [155]. Interestingly, the miR-146a polymorphism (rs2910164 C > G) increased the risk of CHD among non-smokers and hypertensive patients [156].

FLAP expression is also regulated by miR-135a and miR-199a-5p. Overexpression of anti-miR-135a and anti-miR-199a-5p oligonucleotides was shown to result in multiple increases in FLAP mRNA and protein expression [142,157].

In addition to participation in the regulation of lipid mediator production, microRNAs can regulate reverse cholesterol transport (Figure 2). miR-33a and miR-33b inhibit ABCA1 expression [158]. In mouse macrophages, miR-33 inhibits ABCG1 expression, reducing cholesterol efflux into nascent HDL [159,160]. Inhibition of miR-33 in mouse experiments, increases levels of ABCA1 and circulating HDL, suggesting an atherogenic role for miR-33 [161]. Inhibition of miR-33a/b in nonhuman primates increases hepatic expression of ABCA1, increases plasma HDL levels, and decreases triglyceride levels in very low-density lipoproteins. [162,163].

miR-144-3p also targets ABCA1, leading to decreased HDL cholesterol levels. Agomir of miR-144-3p has been shown to effectively accelerate atheromatous plaque formation in *ApoE^−/−^* mice, impairing reverse cholesterol transport and stimulating production of the proinflammatory cytokines TNF-α, IL-1β, and IL-6 [164]. The clinical significance of these data is supported by the positive correlation of circulating miR-144-3p concentration with serum creatine kinase, creatine kinase-MB fraction, lactate dehydrogenase, and aspartate aminotransferase in patients with acute myocardial infarction, allowing miR-144-3p to be considered as a potential candidate biomarker of acute myocardial infarction [164].

ABCA1 has been demonstrated to be a direct target of miR-30e and miR-92a [165]. A negative correlation was found between plasma ABCA1 levels and plasma exosomal levels of miR-30e, suggesting diagnostic potential of miR-30e levels in exosomes as a biomarker of coronary atherosclerosis [165]. MiR-92a, is a component of the miR-17-92 cluster and is highly expressed in human endothelial cells, where it controls angiogenesis [166]. MiR-92a has also been shown to contribute to cardiovascular disease in diabetes via NF-κB and subsequent inflammatory pathways [167].

Endothelial miR-92a is a regulator of Kruppel-like factor 4 (KLF4) biogenesis and also plays a role in KLF2 expression, which demonstrates cross-links with hemodynamic factor effects [168,169,170,171,172,173]. The expression of miR-92a is increased in endothelial cells and blood flow in atherosclerosis. It was also found that endothelial miR-92a can be transported to macrophages mainly through extracellular vesicles, where, by regulating KLF4 levels, it promotes their atherogenic phenotypic switching, inflammatory activation, and increased LDL uptake [174]. In addition, miR-92a can bind to HDL in the serum of vulnerable patients with CHD [175]. These data reinforce the understanding of the importance of miR-92a as an important participant in the provision of arterial homeostasis [174]. Levels of miR-92a-3p were significantly elevated in patients with CHD, with endothelial cells being a major source of cells for microvesicles containing miR-92a-3p. EMV-mediated functional miR-92a-3 transport regulates angiogenesis and is an important tool for intercellular communication [176]. MiR-92a is known to regulate the expression of KLF4 and KLF2 in arterial endothelium. Inhibition of miR-92a prevents endothelial dysfunction and atherosclerosis in mice [177]. This is because miR-92a regulates oxLDL-mediated endothelial cell activation under low shear voltage conditions, which has been linked to KLF2 and KLF4 modulation and suppresses cytokine signaling [177]. It was shown that miR-92a knockdown in vitro led to a partial reduction in the expression of proinflammatory markers induced by cytokines, which was associated with enhanced expression of KLF4 [168].

It was also shown that circulating miR-92a could be associated with the HDL3 fraction, and their levels differed in stable and vulnerable patients with CHD [175]. Thus, HDL are involved in an intercellular communication mechanism involving microRNA transport and delivery [178]. Of interest is the evidence that healthy HDL, through clathrin-mediated endocytosis, is taken up by macrophages and attenuates 5-LOX expression by the ubiquitin proteasome system, resulting in reduced LTB4 from activated macrophages [179]. This mechanism is another lipid-associated pathway of inflammation regulation.

## 5. The Significance of Long Non-Coding RNAs

Long non-coding RNAs (long ncRNAs, lncRNAs) are another important regulatory link whose clinical significance is only beginning to be understood. Most of the human genome is known to be transcribed into noncoding RNAs. lncRNAs are a type of RNA, more than 200 nucleotides long, that are not translated into protein [180]. However, they modulate gene expression and are involved in a variety of pathophysiological processes [181,182,183]. Several lncRNAs have been identified as epigenetic regulators involved in the development of cardiovascular disease.

Seven lncRNAs (RP11-68I3.11, AC068831.6, RP11-133L14.5, PAX8-AS1, RP11-259K15.2, RP11-203M5.8, and LINC01254) have been identified in patients with acute coronary syndrome and may be potential biomarkers of myocardial infarction [184]. In addition, circulating ENST00000538705.1 has been found to facilitate the progression of acute coronary syndrome through modulation of *ALOX15* [185]. Knockdown of ENST00000538705.1 or *ALOX15* reduces myocardial damage, decreases serum total cholesterol and LDL levels, and increases HDL levels in rats with myocardial infarction. This allows ENST00000538705.1 and *ALOX15* to be considered as potential molecular targets for acute coronary syndrome therapy [185].

It has been shown that lncRNAs are found in the border zone of myocardial infarction in rats. The same study showed that lncRNA AY212271 is coexpressed with *Alox5ap* and can participate in the inflammatory response in the border zone of myocardial infarction indirectly through *Alox5ap* [186].

In addition, lncRNAs are involved in the regulation of reverse cholesterol transport, thereby influencing the development of atherosclerosis. ABCA1 has previously been found to be regulated by various lncRNAs, including MeXis, GAS5, TUG1, MEG3, MALAT1, Lnc-HC, RP5-833A20.1, LOXL1-AS1, CHROME, DAPK1-IT1, SIRT1 AS lncRNA, DYNLRB2-2, DANCR, LeXis, LOC286367, and LncOR13C9. ABCG1 is also regulated by several lncRNAs, such as TUG1, GAS5, RP5-833A20.1, DYNLRB2-2, ENST00000602558.1, and AC096664.3 [187,188].

In addition to the fact that TUG1 expression reduces macrophage cholesterol export by mediating the expression of ABCA1 and ABCG1, it can also reduce ApoM expression in the miR-92a/ FXR1 axis [189]. These cross-linkages are of interest because TUG1 overexpression in *ApoE^−/−^* mice can increase plaque size and consequently increase macrophage cholesterol content. It has been shown that TUG1 can bind miR-92a while competitively inhibiting FXR1 and miR-92a binding. This stimulates FXR1 to further reduce ApoM expression. In addition, TUG1 can enhance the inflammatory response in the plasma, which is characterized by increased inflammatory cytokines, such as TNFα, IL-1β, and IL-6, as well as impaired cholesterol export [189]. Given that miR-92a promotes atherosclerosis by targeting ABCA1, it is suggested that TUG1 may compete with ABCA1 for binding to miR-92a to reduce ABCA1 expression.

Interestingly, lincRNA DYN-LRB2-2 can promote ABCA1-mediated cholesterol efflux by reducing TLR2 expression in macrophages. Meanwhile, overexpression of TRL2 reverses the effects of lincRNA-DYNLRB2-2 on cholesterol efflux and ABCA1 expression levels [190]. This represents another interesting mechanism of cross-linking inflammation and reverse cholesterol transport.

In turn, lincRNA MALAT1 can target miR-17-5p to regulate the expression level of ABCA1. MALAT1 knockdown increases ox-LDL uptake and promotes cholesterol accumulation by regulating the miR-17-5p/ABCA1 axis in ox-LDL-induced THP-1 macrophages [191]. lincRNA PCA3 inhibits lipid accumulation and atherosclerosis by promoting ABCA1-mediated cholesterol efflux for by sponging miR-140-5p and enhancing RFX7 [192]. Thus, the study of potential targets for lincRNA is of research and clinical interest.

## 6. The Significance of circRNAs

Circular RNAs (circRNAs) are single-stranded noncoding RNAs with a closed ring structure without 3′ or 5′ ends and with binding sites for miRNAs. CircRNAs are important post-transcriptional regulators of gene expression. They act as decoys for microRNA, thereby reducing their ability to target mRNAs [193,194]. Through this targeting and suppression of microRNA activity, circRNAs perform various biological functions and may be involved in the pathogenesis of many diseases, including atherosclerosis [194,195,196,197].

It has been shown that circDENND1B could sponge miR-17-5p and increase Abca1 expression, promoting cholesterol efflux and inhibiting atherogenesis [198]. Another circ_0001445 ring RNA in an experiment on human primary aortic endothelial cells reduced ox-LDL-induced endothelial damage by acting as a sponge miR-208b-5p and thereby regulating ABCG1 [199]. In addition, miR-33a, which regulates ABCA1 expression, was identified as a direct target for circFASN [200].

Thus, circRNAs are an important link in the regulation of the expression of genes related to lipid metabolism and atherosclerosis, which is a promising topic for future re-search.

## 7. Conclusions

The significance of genetic factor in the development and progression of atherosclerosis has yet to be assessed, but a growing body of evidence is increasing the understanding of the role of gene polymorphism and epigenetic regulation in the processes associated with inflammation in the vascular wall, lipid metabolism, and hemodynamic regulation. Lipid metabolism and the innate immune system have been closely linked throughout the natural history of atherogenesis. Significant data support evidence for the important regulatory role of microRNAs. The identification of their targets and cross-linkages is an important and promising topic for further research. MicroRNAs can act on multiple targets, highlighting the complexity and interdependence of processes occurring in the vascular wall. Additionally of interest is the cross-linking of 5-LOX and microRNAs on one side as a target and on the other as a regulator of their production. These cross-links are a potentially important regulatory mechanism. lncRNAs represent another important target for research. Their potential role in atherogenesis is still largely unknown, but available data show significant clinical promise.

Post-transcriptional regulation of 5-lipoxygenase mRNA expression through alter-native splicing and nonsense-mediated mRNA decay is also of great interest [201]. Alternative splicing leads to the formation of several identified 5-LOX isoforms, representing a mechanism for the regulation of the 5-LOX pathway and lipid mediator biosynthesis [202].

These and other data reinforce the focus on identifying the genetic basis of atherogenesis. A growing body of evidence suggests complex cross-linkages in immune and metabolic processes, as well as multiple links in the regulation of gene expression in the complex chain of processes leading to atherosclerosis. The study of these links can con-tribute to a better understanding of the clinical aspects of atherosclerosis, improve the quality of diagnosis and treatment efficacy.

## Figures and Tables

**Table 1:** Some pathways of regulation of cellular cholesterol homeostasis in macrophages.

Factors of Cellular Homeostasis of Lipids in Macrophages	Mechanisms	Participants	Regulation	References
Promote cholesterol accumulation	Phagocytosis/efferocytosis; Lipid uptake; Cholesterol biosynthesis	Modified low density lipoprotein (LDL); scavenger receptors (SR-A, CD-36, LOX-1); HMG-CoA reductase	MicroRNAs, lncRNAs, circRNAs, secondary messengers such as cyclic AMP, nuclear receptors (e.g., LXR, RXR, PPAR and PXR), SREBPs, cytokines, hormones, post-translational modifications, proteasomal, lysosomal, and calpain systems	[102,103,104,105,106,107]
Promote cholesterol efflux	Reverse cholesterol transport	ABCA1 ABCG1 SR-BI ApoAI HDL		

## Data Availability

Not applicable.
